# Real-time prediction of Poisson’s ratio from drilling parameters using machine learning tools

**DOI:** 10.1038/s41598-021-92082-6

**Published:** 2021-06-15

**Authors:** Osama Siddig, Hany Gamal, Salaheldin Elkatatny, Abdulazeez Abdulraheem

**Affiliations:** 1 Department of Petroleum Engineering, King Fahd University of Petroleum and Minerals, Box 5049, Dhahran, 31261 Saudi Arabia; 2 Petroleum Department, Cairo University, Giza, Egypt

**Keywords:** Energy science and technology, Scientific data

## Abstract

Rock elastic properties such as Poisson’s ratio influence wellbore stability, in-situ stresses estimation, drilling performance, and hydraulic fracturing design. Conventionally, Poisson’s ratio estimation requires either laboratory experiments or derived from sonic logs, the main concerns of these methods are the data and samples availability, costs, and time-consumption. In this paper, an alternative real-time technique utilizing drilling parameters and machine learning was presented. The main added value of this approach is that the drilling parameters are more likely to be available and could be collected in real-time during drilling operation without additional cost. These parameters include weight on bit, penetration rate, pump rate, standpipe pressure, and torque. Two machine learning algorithms were used, artificial neural network (ANN) and adaptive neuro-fuzzy inference system (ANFIS). To train and test the models, 2905 data points from one well were used, while 2912 data points from a different well were used for model validation. The lithology of both wells contains carbonate, sandstone, and shale. Optimization on different tuning parameters in the algorithm was conducted to ensure the best prediction was achieved. A good match between the actual and predicted Poisson’s ratio was achieved in both methods with correlation coefficients between 0.98 and 0.99 using ANN and between 0.97 and 0.98 using ANFIS. The average absolute percentage error values were between 1 and 2% in ANN predictions and around 2% when ANFIS was used. Based on these results, the employment of drilling data and machine learning is a strong tool for real-time prediction of geomechanical properties without additional cost.

## Introduction

Rock elasticity is a major identifier for rock mechanical properties and reflects the ability of the rock to recover from a deformation caused by external forces. Two main properties are used to define rock elasticity, Young’s modulus, and Poisson’s ratio. These geomechanical properties show the relationship between the forces and the resulted deformation^1^. Young’s modulus (E) is a stiffness measure and defined by the ratio between the strain and the stress. While Poisson’s ratio (ν) is the ratio between lateral and longitudinal strain (ε). Rock elastic properties influence hydraulic fracturing design, drilling performance, in-situ stresses estimation, and wellbore stability^2–5^.

In order to estimate Poisson’s ratio, there are two options, using core samples or well logs. The Poisson’s ratio determined by compressional tests on core plug samples is called static Poisson’s ratio, while the dynamic Poisson’s ratio is derived from shear and compressional acoustic wave velocities logs^6^ using the following equation.

1 $$ \upnu _{{{\text{dyn}}}}  = \frac{{{\text{V}}_{{\text{P}}}^{2}  - 2{\text{V}}_{{\text{s}}}^{2} }}{{2({\text{V}}_{{\text{P}}}^{2}  - {\text{V}}_{{\text{s}}}^{2} )}}, $$

where $\upnu _{{{\text{dyn}}}}$ is the dynamic Poisson’s ratio, V_S_ and V_P_ are the shear and compressional wave velocities respectively.

The advantage of $\upnu _{{{\text{dyn}}}}$ over $\upnu _{{{\text{static}}}}$, is that it can provide a continuous profile, In addition, getting core samples are expensive and time-consuming. To overcome the fact that static and dynamic values for Poisson’s ratio are usually different from each other, many researchers presented empirical correlations between static and dynamic Poisson’s ratio based on linear regression^7–9^. However, some of these correlations are developed using limited samples and for a specific type of formation as summarized in Table 1.

**Table 1:** Different empirical correlations between static and dynamic Poisson’s ratio.

Equation	R^2^	Data points	Rock types	References
$\upnu _{{{\text{st}}}} = 0.71\upnu _{{{\text{dyn}}}} + 0.063$	0.543	8	Limestone, gypsum, basalts, granite, phonolite, andesite	^7^
$\upnu _{{{\text{st}}}} = {\text{aV}}_{{\text{s}}} + {\text{b}}$ $\upnu _{{{\text{st}}}} = {\text{cV}}_{{\text{p}}} + {\text{d}}$ a, b ,c and d vary with rock type	0.22–0.84	130+	Peridotite, granite, pyrite, pyrrhotite	^8^
$\upnu _{{{\text{st}}}} = {\text{a}}\upnu _{{{\text{dyn}}}} - {\text{b}}$ a and b are different for different porosity ranges	0.70–0.92	18	Tight sandstone, siltstone	^9^

While ${\upnu}_{\mathrm{st}}$ is the static Poisson’s ratio, ${{\upnu }}_{\mathrm{dyn}}$ is the dynamic Poisson’s ratio, ${\mathrm{V}}_{\mathrm{p}}$ and ${\mathrm{V}}_{\mathrm{s}}$ are the compressional and shear wave velocities respectively.

Artificial intelligence (AI) has a wide range of engineering, medical and industrial applications^10–12^. The use of machine learning in the oil industry is fast growing in various sectors. These applications include but are not limited to estimation and optimization of drilling parameters^13–18^, drilling fluid properties^19–21^, reservoir fluid properties^22–27^, petrophysical properties^28–32^, and geomechanical properties^33–36^. Different models between static and dynamic Poisson’s ratio were developed using different machine learning methods such as an artificial neural network (ANN), Fuzzy Logic (FL), Functional Network (FN), and Alternating Conditional Expectation (ACE) as presented in Table 2.

**Table 2:** Different correlations for static Poisson’s ratio developed using AI.

Input parameters	Data points	Formation	R^2^	Methods	References
V_S_, V_P_, bulk density	77	NA	0.828	ANN, FL, FN	^37^
Depth, porosity, In-situ stresses, pore pressure, bulk density, V_S_, V_P_	602	NA	0.994	ACE	^38^
V_S_, V_P_	550	Limestone	0.97	ANN	^39^
bulk density, V_S_, V_P_	610	Carbonate	0.97	ANN	^40^
V_S_, V_P_	75	Carbonate	NA	ANN, FL	^41^
bulk density, V_S_, V_P_	692	Sandstone	0.93	ANN	^42^
Gamma-ray, bulk density, porosity, V_S_, V_P_	580	Carbonate	0.97	FN	^43^

Even though these presented models give good correlations between predicted and actual static Poisson’s ratio, but they still require the availability of the shear and compressional velocities, which are used to estimate dynamic Poison’s ratio, and may not always be available. Therefore, correlating between Poison’s ratio and drilling parameters, which are available from the first encounter to the well, will be extremely beneficial. Moreover, successful applications of using the drilling data to obtain information that usually requires logs have been reported, namely bulk density and sonic velocity logs^44,45^. Furthermore, the use of drilling data in the estimation of formation pressure and abnormal pressure zones detection is an old technique^46,47^.

The approach presented in this paper is based on the idea that drilling data are always available, easier and earlier to obtain compared to conventional well logs and core samples. The use of drilling parameters for real-time estimation of Poisson’s ratio using different AI techniques is investigated and presented in this paper.

## Data and methods

**Figure 1:**
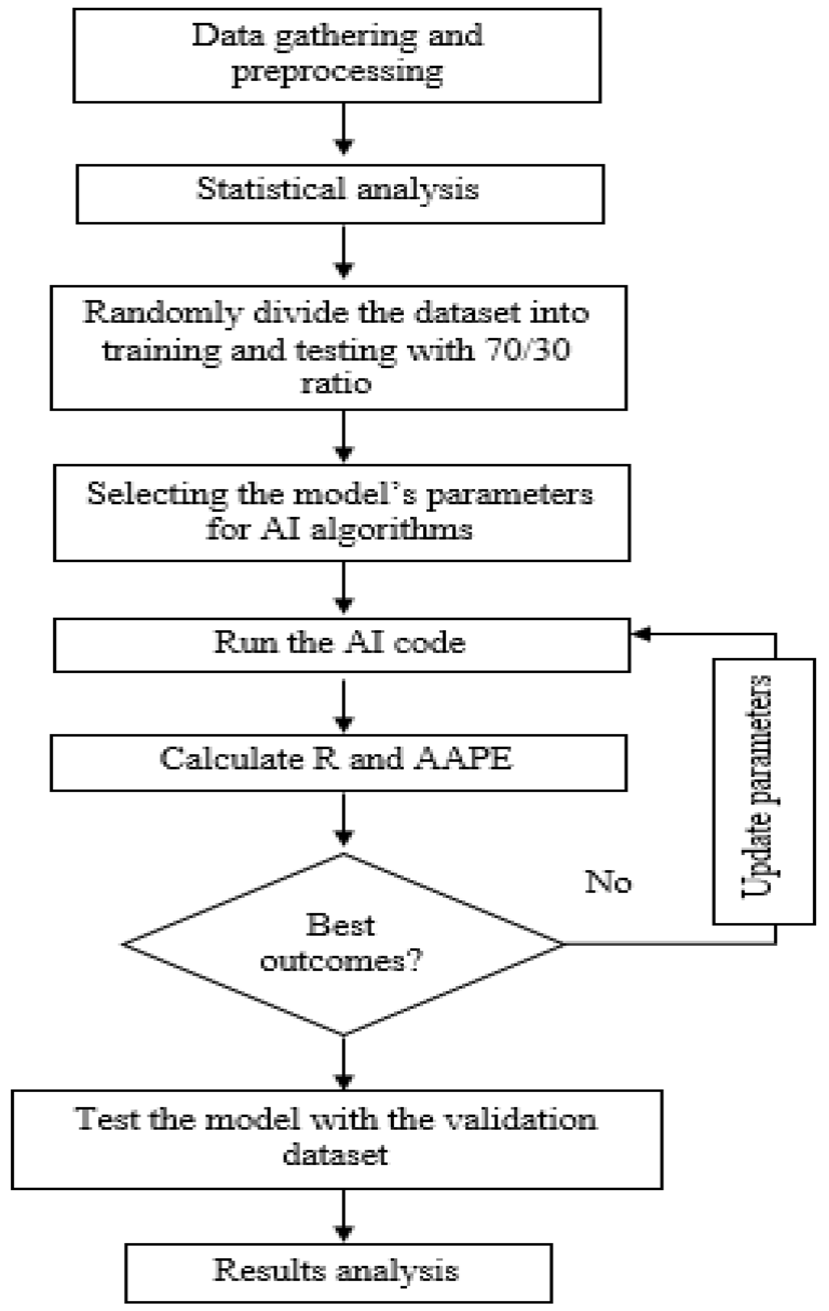
Flow chart for the methodology used to generate AI-model.

In order to predict Poisson’s Ratio from the drilling parameters, the following steps have been followed. Data for drilling parameters and Young’s modulus have been gathered for two wells. Data from one well, has been used to build the model using several machine learning techniques. The dataset from the second well has been hidden from the algorithms and not used later to validate the built model. Figure 1 summarizes the methodology used for efficient young’s modulus prediction.

### Data description

**Figure 2:**
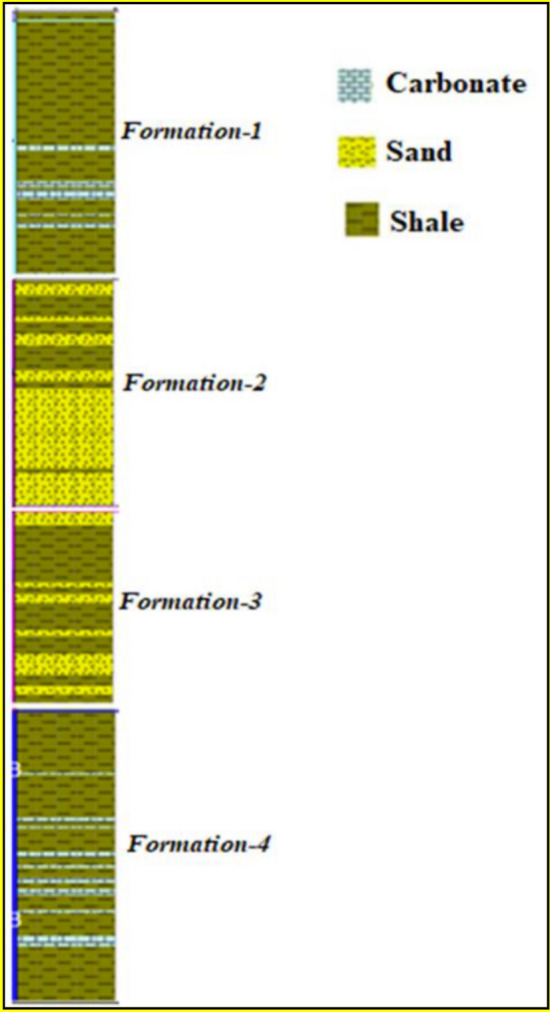
Lithology column for Well-1.

The collected data for this study were gathered from drilling phase activities in the Middle East. The data covered the drilling parameters and the relevant Poisson’s ratio values during drilling the intermediate section for 12.25″ hole size for vertical profile wells. As shown in Fig. 2, the complex lithology of the drilled formations through Well-1 covered four formation types (shale, sandstone, and carbonate rocks).

Well-1 has a total of 2905 data points used to build the model with 70% of the data points for training and 30% for testing the model. 2912 data points from well-2 were hidden from the AI algorithms and used later to validate the built model. Besides the PR that is set as targeted output, each data point contains six drilling parameters used as inputs. The drilling parameters, listed below, were obtained from field measurements and used in building this model:

- Weight on bit WOB in klb
- Torque in kft.lbf
- Standpipe pressure SPP in psi
- Rotary speed RPM (1/min)
- Drilling rate of penetration ROP in ft/h
- Drilling fluid flow Rate in gpm

### Data analysis

Before running the data into the machine learning algorithms, the datasets were cleaned from noise and outliers using Matlab code. Statistical analysis of the dataset used to build the models is presented in Table 3.

**Table 3:** Statistics of the data used to build the models.

	WOB	Torque	SPP	RPM	ROP	Flow rate	PR
Minimum	1.94	4.42	2237.00	92.43	35.00	639.07	0.21
Maximum	24.12	10.66	3008.00	159.65	108.35	852.61	0.42
Mean	12.02	7.45	2606.09	128.97	66.03	728.48	0.33
Median	11.29	7.05	2615.30	134.80	70.00	700.29	0.33
Skewness	0.23	0.21	− 0.13	− 0.51	0.20	0.49	0.15
Coefficient of variation	0.58	0.23	0.07	0.12	0.26	0.10	0.12
Standard deviation	7.00	1.74	192.73	15.00	17.33	73.92	0.04

**Figure 3:**
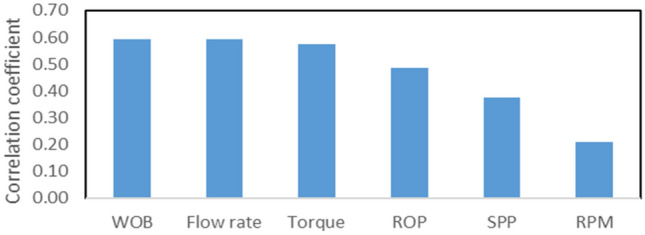
The correlation coefficient of drilling parameters with Poisson’s ratio.

The correlation coefficients between PR and different drilling parameters are given in Fig. 3. It shows relatively strong correlations between PR and some drilling parameters such as WOB, torque, and pump flow rate. Lower correlation coefficients for other parameters don’t necessarily imply the absence of relation between these inputs and PR, but rather means that the linear equation doesn’t describe the relationship between the inputs and the output.

### Machine learning algorithms

For the purpose of constructing the models between Poisson’s ratio and drilling parameters, two machine-learning methods were used separately, artificial neural network (ANN) and adaptive neuro-fuzzy inference system (ANFIS). ANN is a very common machine-learning tool that is inspired by biological neurons in brains^48^. ANN could function as supervised or unsupervised machine learning in regression, classification, and clustering problems^49^. ANN is composed of different components such as neurons, transfer functions, training functions, learning functions, and hidden layers^37^. In literature, there are many reported successful applications of ANN in the oil and gas industry^32,35,36,50,51^.

Adaptive neuro-fuzzy inference system (ANFIS) was developed in the 1990s and integrates the principles of neural networks and fuzzy logic (FL)^52,53^. In this method, ANN is used to set the fuzzy rules in FL^54^. This integration of the two methods provides an improved performance^55^. Similar to ANN, ANFIS has various reported applications in the oil industry^56–59^.

### Models evaluation

ANN and ANFIS were used for models' construction. These algorithms use 70% of the dataset from well-1 to build the model and 30% of the data to test it internally for several iterations and chose the best fit. After having the model, data from well-2 were used as an external validation set for the models. To evaluate all models' trials, two statistical parameters were used, correlation coefficient (R) and average absolute percentage error (AAPE). R and AAPE are calculated using Eqs. (2) and Eq. (3):

2 $$ R = \frac{{\left[ {N\mathop \sum \nolimits_{{i = 1}}^{N} \left( {\nu _{{given~i}}  \times \nu _{{Predicted~i}} } \right)} \right] - \left[ {\mathop \sum \nolimits_{{i = 1}}^{N} \nu _{{given~i}}  \times \mathop \sum \nolimits_{{i = 1}}^{N} \nu _{{Predicted~i}} } \right]}}{{\sqrt {\left[ {N\mathop \sum \nolimits_{{i = 1}}^{N} \left( {\nu _{{given~i}} } \right)^{2}  - \left( {\mathop \sum \nolimits_{{i = 1}}^{N} \nu _{{given~i}} } \right)^{2} } \right]\left[ {N\mathop \sum \nolimits_{{i = 1}}^{N} \left( {\nu _{{Predicted~i}} } \right)^{2}  - \left( {\mathop \sum \nolimits_{{i = 1}}^{N} \nu _{{Predicted~i}} } \right)^{2} } \right]} }} $$

3 $$ AAPE = \frac{{\mathop \sum \nolimits_{{i = 1}}^{N} \frac{{\nu _{{given~i}}  - \nu _{{Predicted~i}} }}{{\nu _{{given~i}} }} \times 100\% }}{N} $$

where ${\nu }_{given}$ and ${\nu }_{Predicted}$ are the available and the predicted Poisson’s ratio respectively, and N is the total number of data points.

### Sensitivity and optimization

Different runs were done in each method to determine the best tuning parameters inside the algorithms. This has been done by running the two machine learning methods inside multiple for-loops containing the range of tested parameters. In ANN models, a different number of neurons, network functions, training functions, and transfer functions were used. In ANFIS, different cluster radiuses and the number of iterations were used. Table 4 shows the total range of parameters used to get the best models.

**Table 4:** Different parameters used to optimize the models.

Number of neurons	ANN			ANFIS	
	Network functions	Training functions	Transfer Functions	Cluster radius	Number of iterations
5	fitnet	trainbr	logsig	0.3	100
10	newcf	trainlm	hardlims	0.5	300
15	newelm	trainbfg	poslin	0.7	
35	newlrn	traincgb	purelin	0.9	
45	newp	traincgf	hardlim		
	newdtdnn	traincgp	radbas		
	newff	traingda	satlins		
	newfftd	traingdx	compet		
	newfit	trainoss	netinv		
	newnarx	trainrp	satlin		
	newnarxsp	trainscg	softmax		
	newc	trainb	tribas		
		trainr			

## Results and discussion

### Avoiding overfitting

Overfitting is a very troublesome problem in machine learning, in which the model fits very well in training data and performs poorly in validation and testing. Overfitting results in a model that is limited only to the training data and could not be generalized for data from different sources. In this work, overfitting has been overcome by different methods.

In machine learning, when the number of parameters used to optimize the fitting, such as weights and biases, is too much compared to the number of data points, this will increase the chances of overfitting. As indicated in the data description section, more than 2000 data points were used to train the model, which is relatively a high number. This data quantity helped to improve model generalization. Moreover, the models were built to be as simple as possible. For instance, in ANN one layer of neurons was used and the number of neurons was chosen to be as less as possible without significantly affecting the fitting performance.

**Figure 4:**
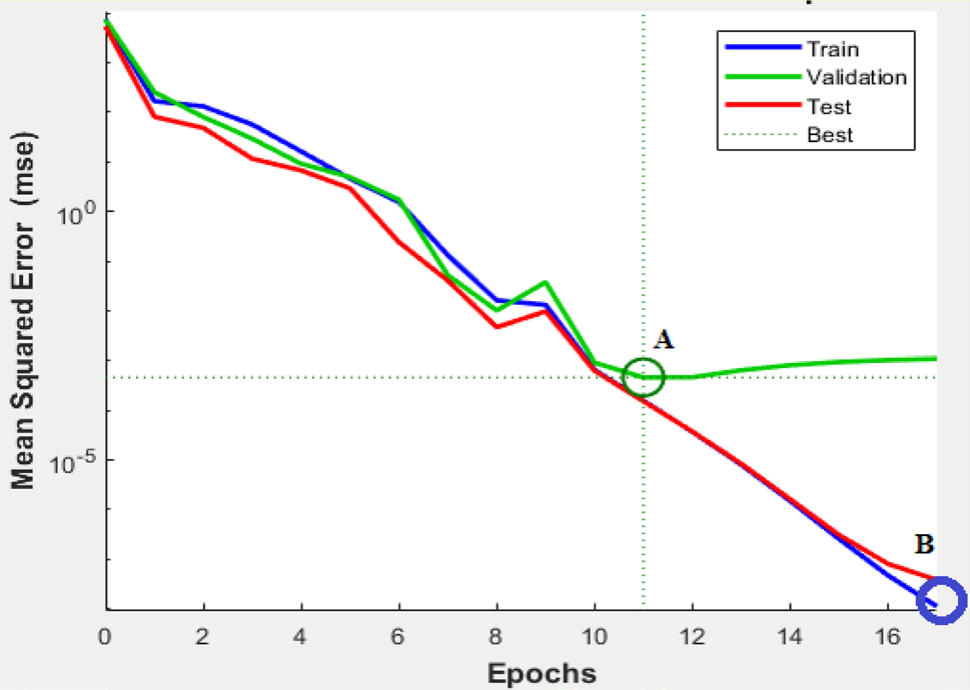
Early stopping to avoid overfitting.

Additionally, the used algorithms have an early stopping feature to avoid overfitting. In this feature, part of training data is separated and will not be used to build the model instead it will be used as an early validation. The fitting performance for training and validation is estimated at each iteration. For each iteration in Fig. 4, both training and validation error is reducing till point A, after which the model starts to overfit and the validation error starts to increase. Due to the early stopping feature, point A parameters will be used in the model instead of point B, even though it has less error in training.

### Artificial neural network

#### Sensitivity

**Figure 5:**
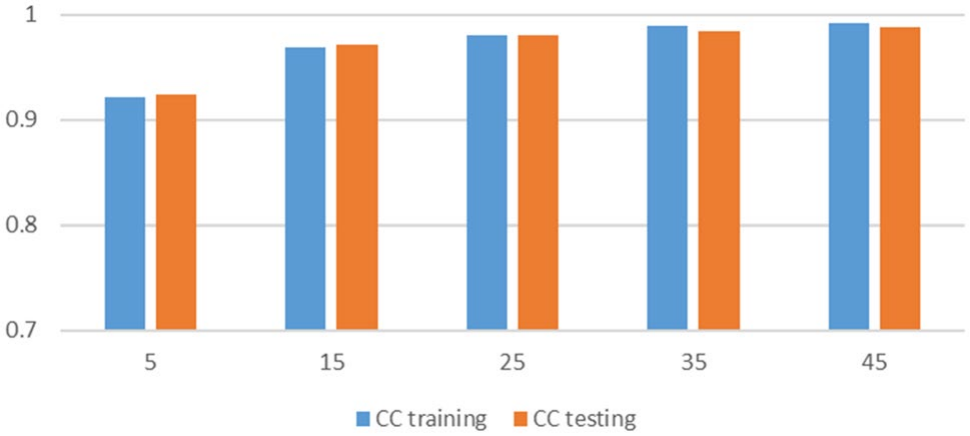
Sensitivity analysis on the number of neurons.

**Figure 6:**
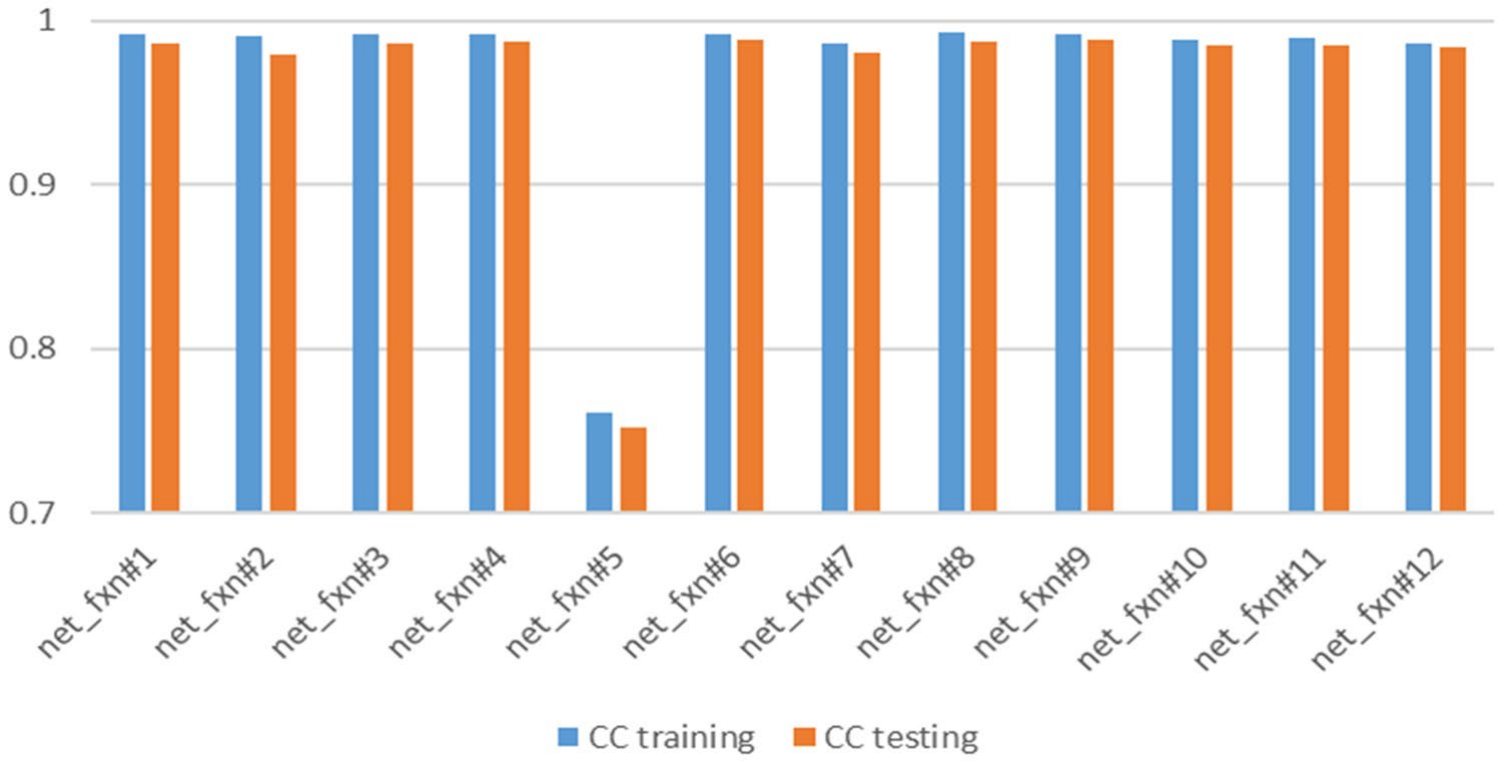
Sensitivity analysis on different network functions.

**Figure 7:**
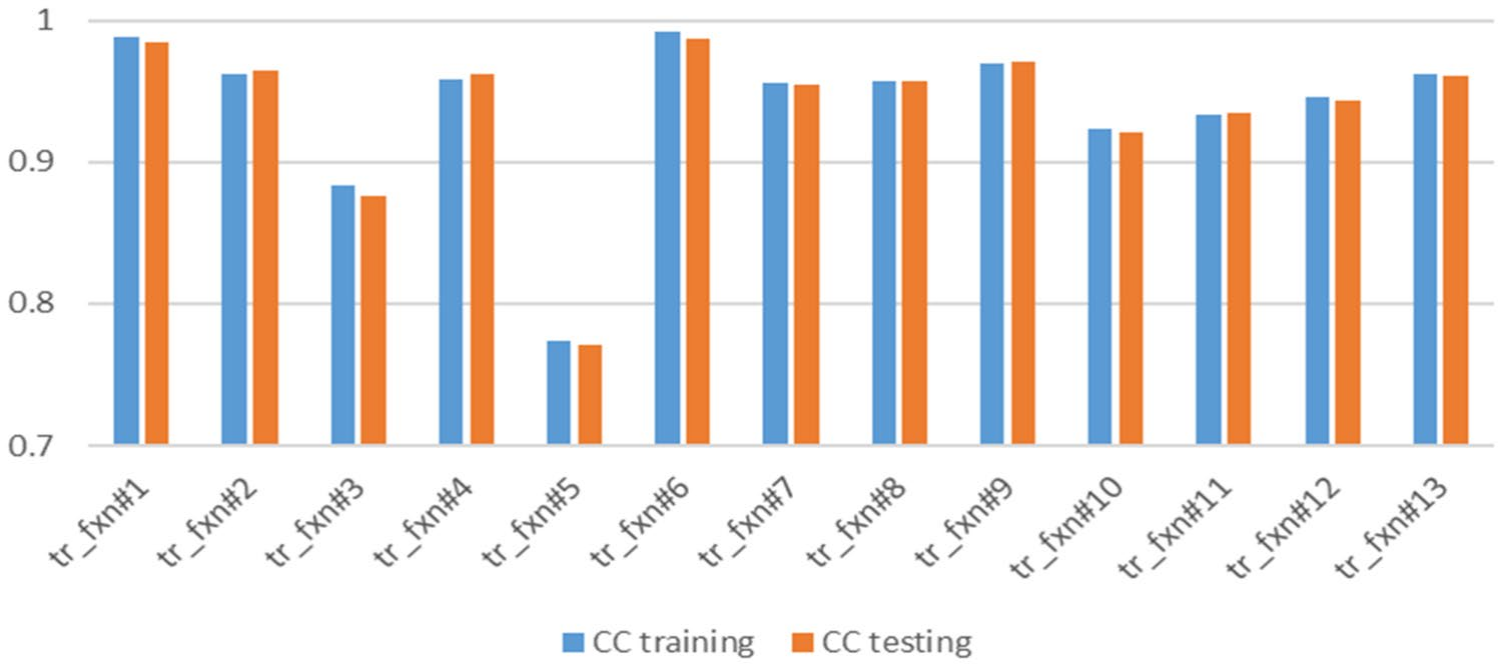
Sensitivity analysis on different training functions.

**Figure 8:**
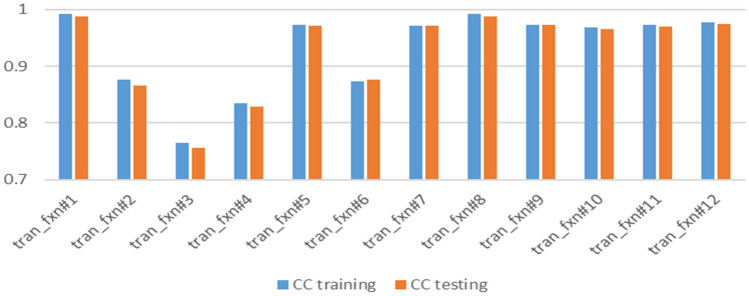
Sensitivity analysis of different transfer functions.

To ensure the best results from ANN, a different number of neurons, network functions, training functions, and transfer functions were used. Figures 5, 6, 7 and 8 present the sensitivity analysis on these parameters. Increasing the number of neurons results in better results, however, the computational time increases as well. In addition, there is no significant increase in correlation coefficients when more than 25 neurons were used as shown in Fig. 5. Except for one case, there were no significant variations when different network functions were used as demonstrated in Fig. 6. Sensitivity analyses on training and transfer functions showed the most variations with correlations coefficient ranging between 0.75 and 0.99 as illustrated in Figs. 7 and 8.

#### Validation

**Figure 9:**
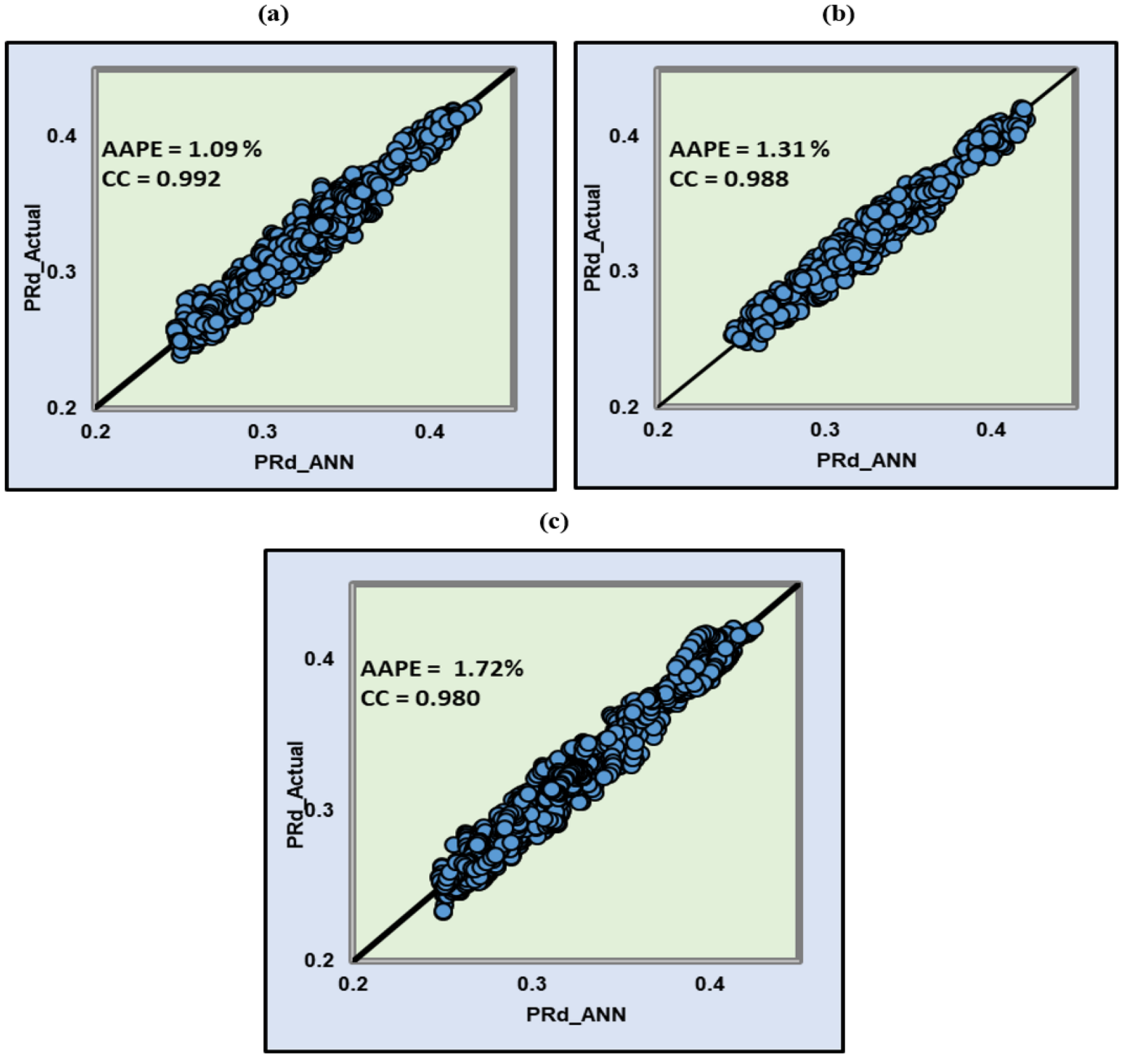
Actual and ANN predicted Poisson’s ratio for (**a**) training (**b**) testing and (**c**) validation.

The dataset from well-1 was used to build the model and to perform the sensitivity analysis. After the model has been built, data from well-2 have been used to validate the model. Good results have been achieved in both wells even though the algorithm only trained and test the model using the first well data. The correlation coefficients were 0.992, 0.988 and 0.980 for training, testing, and validation respectively, and the AAPE values were all in the range between 1 and 2%. Figure 9. Shows a comparison between actual and ANN predicted Poisson’s ratio for well-1 and well-2.

### Adaptive neuro-fuzzy inference system

#### Sensitivity

**Figure 10:**
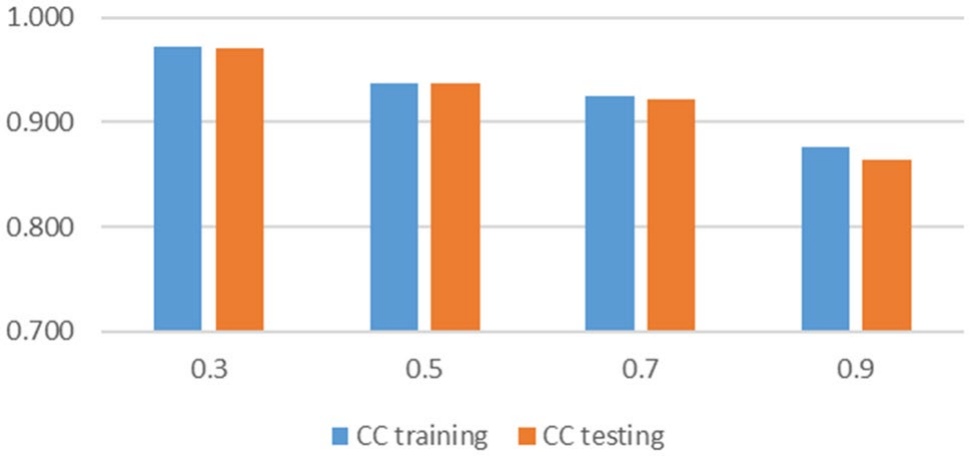
Sensitivity analysis on cluster radius.

**Figure 11:**
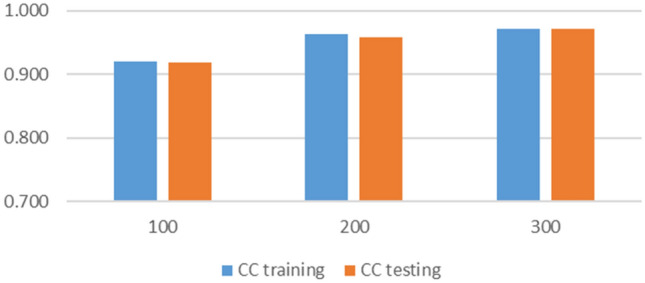
Sensitivity analysis on the number of iterations.

Using ANFIS, different cluster radiuses and number of iterations were used. Sensitivity analysis of these two parameters is presented in Figs. 10 and 11. Increasing the cluster radius from 0.3 to 0.9 resulted in a decrease in correlation coefficients from 0.97 to 0.88 in training and from 0.97 to 0.86 in testing. On the other hand, increasing the number of iterations enhanced the results.

#### Validation

**Figure 12:**
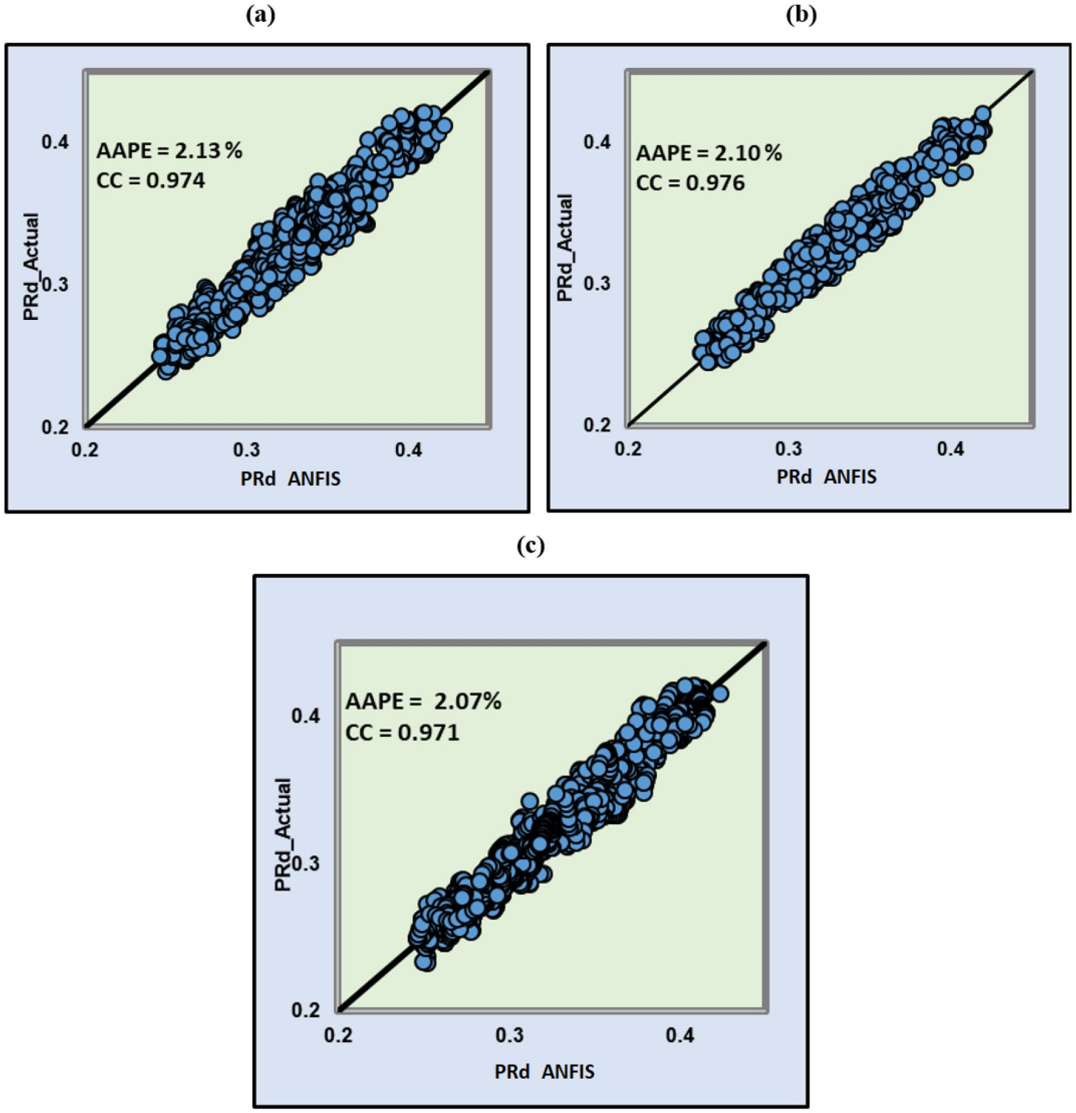
Actual and ANFIS predicted Poisson’s ratio for (**a**) training, (**b**) testing and (**c**) validation.

The same procedure used in ANN has been used in the ANFIS model's building and validations. The data set from Well-1 have been used to train and test the model using different parameters and Well-2 dataset was used to validate the built model. Even though all correlation coefficients were higher than 0.97 and the AAPE values were less than 2.2%, the ANN results presented earlier are better. The actual Poisson’s ratio in comparison with the predicted Poisson’s ratio with ANFIS is presented in Fig. 12.

### Computational cost

**Figure 13:**
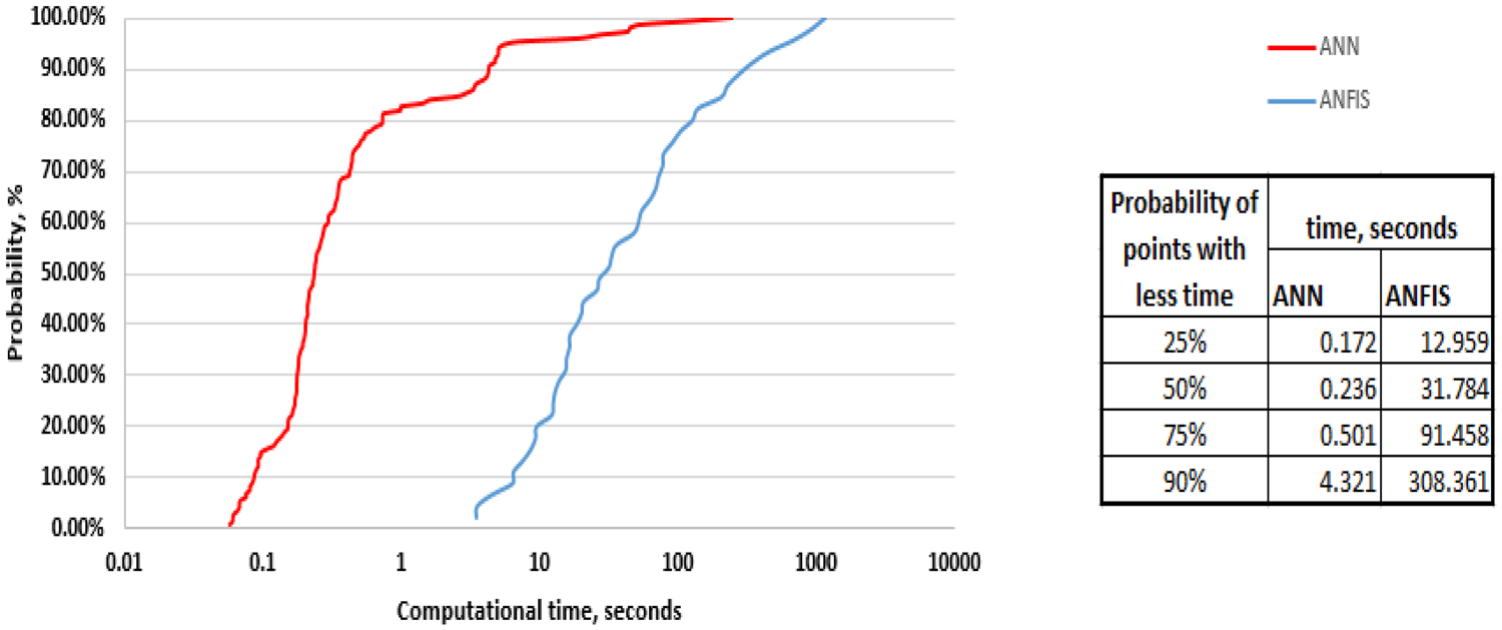
The ascending probability of computational time for the two methods.

Besides the key performance indices (correlation coefficient and average absolute percentage error), the computational cost is considered a very essential factor used to compare the different methods utilized. The calculation times (in seconds) were determined in each run for the two models in order to compare the calculation efficiency. As shown the Fig. 13, ANN outperformed the ANFIS model with 90% of the runs took less than 4.32 s while in ANFIS this value was more than 300 s.

### Model

Different parameters' combinations have been tested to ensure optimum fit. Table 5 displays ANN and ANFIS parameters that yielded the best matches between the predictions and given values.

**Table 5:** Machine learning’s parameters with the best performance.

ANN-parameters	
Number of hidden layers	1
Number of neurons	25
Types of network function	newlrn
Types of training function	Bayesian regularization backpropagation
Types of transfer function	Log-sigmoid transfer function
Maximum number of iterations	1000
Learning Rate	0.12
Momentum constant	0.6
Minimum performance gradient	1.00E−06
Maximum value for mu	1.00E+100

ANFIS-parameters	
Fuzzy inference system	subtractive clustering
Cluster Radius	0.3
Epochs size	300

The best fit was obtained using ANN with a correlation coefficient around 0.99 in training and testing and 0.98 in the validation process and AAPE between 1 and 2%. The generated model is expressed by Eq. 4, while Table 6 shows the weight and biases that are used in the model.

4 $$ \nu _{{dyn}}  = \left[ {\mathop \sum \limits_{{i = 1}}^{N} W_{{2,i}} \left( {\frac{2}{{1 + e^{{ - 2\left( {W_{{11,i}} *WOB + W_{{12,i}} *Torque + W_{{13,i}} *SPP + W_{{14,i}} *RPM + W_{{15,i}} *ROP + W_{{16,i}} *pump~rate + b_{{1,i}} } \right)}} }}} \right)} \right] + b_{2} $$

**Table 6:** Weights and biases (b2 = 0.2).

i	W_11_	W_12_	W_13_	W_14_	W_15_	W_16_	b_1_	W_2_
1	− 1.40	− 0.64	− 0.07	0.26	− 1.03	0.83	− 1.57	− 4.10
2	− 1.11	0.21	− 3.08	− 0.11	0.20	3.32	2.46	− 5.51
3	2.99	− 3.34	3.08	1.91	3.69	− 5.10	− 0.22	0.78
4	− 0.73	0.83	− 2.27	0.79	1.83	− 1.57	0.27	3.26
5	3.47	− 2.79	2.85	0.74	− 3.00	1.30	− 1.04	2.92
6	− 0.89	− 2.18	− 0.46	− 3.71	2.83	− 0.38	1.34	− 2.44
7	− 0.47	− 1.26	0.37	2.05	− 3.75	0.32	− 2.27	1.66
8	− 1.38	− 0.15	− 7.26	3.55	− 2.87	1.93	0.74	0.81
9	3.41	− 2.71	1.72	− 0.60	0.11	1.06	− 0.62	− 4.74
10	− 1.78	− 2.48	− 0.48	− 3.73	3.58	− 0.02	1.14	1.75
11	− 0.47	− 1.16	1.00	2.22	− 3.15	0.33	1.66	1.79
12	2.86	− 0.63	2.47	2.35	− 3.27	0.78	− 1.81	− 2.19
13	− 3.07	− 1.23	0.85	0.79	− 3.84	1.13	− 0.34	− 1.16
14	1.25	0.92	1.54	− 0.93	2.08	0.01	− 2.51	2.85
15	2.90	− 1.50	− 1.89	3.78	− 3.58	3.23	− 1.35	− 1.94
16	− 2.22	3.55	0.93	2.54	− 0.35	1.27	− 1.95	− 1.45
17	4.01	− 2.70	1.20	− 0.73	− 1.88	3.68	− 1.51	3.79
18	− 1.90	4.47	− 2.60	− 1.14	2.69	− 1.98	1.35	− 1.42
19	− 3.00	3.38	− 2.97	1.51	1.37	− 2.40	− 2.66	0.88
20	− 2.35	− 2.91	1.32	2.33	1.81	− 3.42	− 0.88	− 3.55
21	1.23	2.68	− 0.77	− 1.79	− 1.60	3.08	1.01	− 5.25
22	0.11	0.35	1.02	1.01	0.35	− 2.14	− 0.94	− 7.96
23	− 1.48	1.63	− 1.28	− 1.43	− 2.04	0.90	− 0.23	− 1.56
24	− 5.81	1.94	2.16	1.15	− 0.69	2.93	− 0.34	0.81
25	− 1.00	2.74	0.69	− 1.56	− 1.27	1.39	− 0.43	1.98

## Conclusions

Conventionally, Poisson’s ratio is estimated from sonic logs data, which may not always be available. An alternative real-time prediction of Poisson’s ratio from drilling data has been proposed in this paper by employing different machine learning tools. In the light of the presented outcomes, the following statements could be used to conclude the study:

- Compared to other means used to predict Poisson’s ratio, drilling data is more likely to be available at an early stage of the well's life without additional cost and time. Therefore, the prediction of Poisson’s ratio from drilling data will be very beneficial.
- Two machine learning methods were investigated and both yielded a good match, however, a slightly better prediction of Poisson’s ratio was achieved using ANN. The sensitivity and optimization of different parameters used in the algorithms have been presented and the best results were reported.
- The correlation coefficient between the actual and predicted values ranged between 0.97 and 0.99, while the average errors were all less than 2.2%. The best model was presented as a white-box to allow using other datasets.

## Recommendations

Supported by the outcomes presented in this paper that confirm the ability to obtain good predictions of Poisson’s ratio from drilling data, it is recommended to investigated other machine learning methods. Moreover, the use of drilling data in the prediction of other geomechanical properties could be investigated using a similar approach. It is also worthy to mention that the data used in this study are from the same field, therefore, to generate general model data from different sources could be combined and used altogether.

## SI Metric Conversion Factors

1 ft = 0.3048 m.

1 lb = 0.453592 kg.

1 lbf = 4.44822 N.

1 psi = 6894.76 Pa.

1 gal = 0.00378541 m^3^.
